# Editorial: Beyond Th1: Novel concepts in tuberculosis vaccine immunology

**DOI:** 10.3389/fimmu.2022.1059011

**Published:** 2022-11-23

**Authors:** Karin Dijkman, Rhea N. Coler, Simone A. Joosten

**Affiliations:** ^1^ Department of Preclinical Immunology, Janssen Vaccines & Prevention, Leiden, Netherlands; ^2^ Seattle Children’s Research Institute, Center for Global Infectious Disease Research, Seattle, WA, United States; ^3^ Department of Pediatrics, University of Washington School of Medicine, Seattle, WA, United States; ^4^ Department of Global Health, University of Washington, Seattle, WA, United States; ^5^ Department of Infectious Diseases, Leiden University Medical Center, Leiden, Netherlands

**Keywords:** vaccines, tuberculosis, mycobacteria, adaptive immunity, innate immunity, immunology & infectious diseases

2021 marked the 100-year anniversary of *Bacillus Calmette Guerin* (BCG), the only licensed tuberculosis (TB) vaccine to date. BCG protects infants from severe forms of pediatric TB, though fails to reliably protect adolescents and adults from pulmonary TB and is thus ineffective in curbing the ongoing TB epidemic. The 1.5 million TB deaths annually underline that the need for a new, more effective, TB vaccine is urgent.

The development of more effective TB vaccines has been hampered by a lack of knowledge on what protective immunity against TB exactly requires. Historically, vaccine developers have optimized their candidate vaccines towards the induction of peripheral interferon-γ (IFN-γ)-producing T-helper type 1 (Th1) cells. The importance of Th1-responses is supported by the increased susceptibility to mycobacteria in individuals with inborn errors in IFN-γ pathways, as well as the increased risk of TB in HIV patients with low CD4^+^ T-cell counts. However, mounting evidence from animal models, clinical trials, and cohort studies suggest that a Th1 response is not solely sufficient for vaccine-mediated protection.

In recent years, new immune subsets and concepts have been investigated in the context of protection against TB, including donor unrestricted T-cells (DURTs), T-helper 17 cells, and antibodies and B-cells. Also innate immune players, like myeloid suppressor cells or innate lymphoid cells (ILC), may be more pivotal than originally anticipated. Furthermore, the potential of pulmonary, tissue-resident immune responses is increasingly appreciated, and mucosal delivery of vaccines to induce these responses is currently being investigated.

In this Research Topic, we collect articles on vaccine-concepts beyond the induction of peripheral Th1 immunity that add to our knowledge on protective immunity and will advance the field of TB vaccine development.

## Vaccines & current insights in vaccine induced immunity

Larsen et al. summarize the current insights in vaccine-induced immunity, including many subsets different from Th1 CD4^+^ T-cells and highlight that players may differ for vaccines that aim to prevent infection compared to those targeting prevention of disease. Subsets such as natural killer (NK) cells, invariant natural killer T-cell (iNKT), CD8^+^ T-cells, γδ T-cells, neutrophils, myeloid dendritic cell (mDC), alveolar macrophages (AM), mucosal-associated invariant T (MAIT) and B-cells might collaborate to combat against TB at different stages of infection and thus their interaction as well as their timing need to be considered more carefully.

Khan et al. developed a novel multi-epitope subunit vaccine comprised of conserved and experimentally confirmed antigens Rv0058, Rv0101, and Rv3343, following evaluation of predicted helper T-lymphocytes (HTL), cytotoxic T-lymphocytes (CTL), and B-cell epitopes. Through dynamic simulations and molecular docking, the vaccine-receptor complex’s stability and high affinity for a TLR-3 immune receptor were validated. Follow-up will be needed to demonstrate a functional role in preventing TB infection.

The emergence of drug resistance to first-line antibiotics poses a threat to successful treatment, which may be addressed by the development of novel therapeutic vaccines. Bouzeyen and David reviews candidates in the pipeline, which could be used as adjunctive treatment of TB or to prevent relapse following antibiotic treatment.

## Antigen recognition & novel T-cell subsets

For T-cell mediated protection, recognition of the *Mycobacterium tuberculosis (Mtb)*-infected host cell is critical. Lewinsohn et al. discuss how current read-outs of immunogenicity often over-estimate the capacity of vaccine-elicited T-cells to respond to infected target cells. Typically, assessment of vaccine-induced CD4^+^ and CD8^+^ T-cells relies on measurement of antigen-specific, cytokine producing polyfunctional cells, often upon restimulation *in vitro*. However, the authors review several strategies employed by *Mtb* to circumvent recognition *in vivo*, including down-regulation of MHC and production of decoy antigens, and argue for the implementation of clinically refined assays determining the capacity of T-cells to recognize infected cells.

Another feature of *Mtb* is its tendency to aggregate. Rodel et al. describe how aggregated *Mtb* influences macrophage responses. Compared to infection with single or multiple non-aggregated *Mtb*, aggregated *Mtb* leads to a distinct pro-inflammatory transcriptional response, reduced acidification of phagosomes and increased cell death. In addition to contributing to infection spread, this response could modulate the host immune response and circumvent recognition by vaccine-induced T-cells, potentially leading to active disease, cell necrosis, and additional cycles of transmission.

Following vaccination, some T-cells recognize non-protein antigens *via* antigen presenting machinery that is independent of genetic background, known as DURT cells. Following BCG revaccination, James et al report a durably expanded set of TCR-δ clonotypes, while no change in the frequency of total MAIT cells, iNKT cells, germline encoded-mycolyl-reactive (GEM) T-cells, or γδ T-cells, was found. These expanded DURT TCR-δ clones expressed the Vδ2 gene segment that likely recognize similar epitopes, which has implications for defining the immunogenicity of candidate whole cell TB vaccines.

## Antibody responses

Endocrine hormones and *Mtb*-specific antibodies in patients with different stages of TB are described by Tsegaye et al.. Interestingly, levels of endocrine hormones were decreased in patients with active pulmonary or extrapulmonary TB, but normalized over successful therapeutic treatment. Lipoarabinomannan (LAM)- specific IgG, IgM, and IgA, were increased in patients with pulmonary TB whereas IgG1 was increased in individuals with latent TB infection (LTBI). In contrast to the hypothesis of the authors, no association was identified between the endocrine hormone levels and circulating LAM-specific antibodies.

Bitencourt et al. elaborate further on the role of antibodies in protection against TB. In BCG-vaccinated humans, the authors identified the induction of PPD-specific antibodies as well as BCG-specific plasmablasts and memory B-cells, suggesting durable antibody responses. A functional role for these antibodies was demonstrated; serum from vaccinated individuals inhibited outgrowth in a mycobacterial growth inhibition assay (MGIA), and enhanced opsonization and phagocytosis by macrophages, which may contribute to control of mycobacterial growth.

## General summary

Before the advent of COVID-19, TB was the leading infectious disease killer globally. The accelerated development of multiple, highly effective, vaccines in the wake of the COVID-19 pandemic, has incited a push for a similar effort towards a novel, more effective, TB vaccine that provides durable control against *Mtb*. Recent insights, including those included in this Research Topic, argue for functional immune responses that encompass more than the induction of peripheral Th1 immunity.

## Author contributions

All authors listed have made a substantial, direct, and intellectual contribution to the work and approved it for publication.

## Conflict of interest

The authors declare that the research was conducted in the absence of any commercial or financial relationships that could be construed as a potential conflict of interest.

## Publisher’s note

All claims expressed in this article are solely those of the authors and do not necessarily represent those of their affiliated organizations, or those of the publisher, the editors and the reviewers. Any product that may be evaluated in this article, or claim that may be made by its manufacturer, is not guaranteed or endorsed by the publisher.

